# From face-to-face to screen-to-screen: exploring the multifaceted dimensions of digital mental health care

**DOI:** 10.3389/fpsyt.2024.1413127

**Published:** 2024-11-04

**Authors:** Aneela Maqsood, Seema Gul, Touseef Zahra, Nazia Noureen, Amira Khattak

**Affiliations:** ^1^ Department of Behavioral Sciences, National Centre for Research on Suicide Prevention, Fatima Jinnah Women University, Rawalpindi, Pakistan; ^2^ General Studies Department, College of Humanities and Sciences, Prince Sultan University, Riyadh, Saudi Arabia; ^3^ Education Department, Govt. School Rasool Pura, Government of Punjab, Pakistan; ^4^ Marketing Department, College of Business Administration, Prince Sultan University, Riyadh, Saudi Arabia

**Keywords:** mental health, e-health, digital counseling, digital psychotherapy, technology adoption, health services accessibility, therapist-client rapport, telehealth intervention adaptation

## Abstract

**Background:**

With the onset of the COVID-19 pandemic, the world witnessed an unprecedented surge in the adoption of digital platforms across various sectors, including mental health care. In countries such as Pakistan, where traditional face-to-face therapy practices hold social and cultural significance, transitioning to digital therapeutic methods presents a range of unique opportunities and challenges.

**Objectives:**

This research aimed to explore the dynamics, implications, and perceptions surrounding digital therapeutic care within the Pakistani sociocultural context. Given the paucity of literature on this subject in the Pakistani context, this study aims to bridge the evidence gap between trends in digital therapy and localized practices. The main goal was to understand the benefits, issues, and challenges therapists and clients face when adopting digital modes for therapeutic care.

**Method:**

For this study, primary data was gathered from counsellors and therapists using a qualitative in-depth interview guide. Using a thematic analysis approach, key themes were derived from the interviews that provided insights into the experiences and perceptions of the participants.

**Results:**

The study revealed that digital platforms have great potential in breaking down geographical barriers making therapeutic interventions more accessible to a wider demographic. This increased accessibility also brought about a level of comfort for clients as they could access therapy from their familiar surroundings. Among the challenges that needed attention, security and confidentiality stood out, requiring strict measures to safeguard client’s data. The shift also brought to light the diverse range of feedback from clients, which was influenced by factors like age and technological proficiency. Moreover, the digital transition posed challenges for both therapists and clients, with many facing an adjustment period as they transitioned from face-to-face to online sessions. One significant challenge was the perceived difficulty in fostering a deep interpersonal connection in a virtual environment. This was further compounded by the need for therapists to modify traditional therapeutic techniques to fit the online mode.

**Conclusion and implications:**

The study underscores the evolving nature of digital therapy in Pakistan, setting a foundation for further exploration in aligning technology with therapeutic needs, ensuring optimal benefits for clients while preserving the sanctity and efficacy of the therapeutic relationship.

## 1 Introduction

In the vast expanse of human history, the timeless act of communication has continuously evolved. From cave drawings and oral traditions to the written word, and now, in our modern era, to the realm of digital communication, the ways we share and receive information have undergone profound transformations. Central to this narrative is the domain of therapy and counselling, where face-to-face interactions have been the bedrock of therapeutic rapport and healing (1). This paper ventures into a significant paradigm shift in therapeutic communication: the move “*From Face-to-Face to Screen-to-Screen*”.

The advent of the 21st century brought with it unprecedented advancements in technology, not least of which was the rise of digital platforms that started bridging gaps across time and space. As the world became increasingly interconnected, the realm of therapy and counselling also began to witness a gradual incorporation of digital tools (2, 3). These tools were initially considered as extensions of therapy gradually found their own place. Teletherapy, e-counselling and digital therapeutic interventions emerged as alternatives, for individuals who due to several reasons couldn’t access traditional therapeutic settings (4, 5).

The significance of digital interventions in the field of therapy and counseling cannot be understated (4). They offer benefits, such as reaching diverse populations and creating a flexible and less intimidating environment for clients (6, 7). For therapists, these interventions provide opportunities for expanding their client base, flexibility in delivering services and adapting to the rapidly evolving digital world. However, along with these advantages come challenges, such as ensuring the same quality of therapeutic rapport as in physical interventions, addressing concerns of digital security, and navigating the technical intricacies of digital platforms (8, 9).

While the global significance of digital therapeutic interventions has been explored to some extent in existing literature, the Pakistani context presents its own set of challenges and opportunities (10, 11). A nation marked by a rich cultural heritage, diversity, and contrasting socio-economic landscapes, Pakistan has been on a continuous journey of digital transformation (12). The COVID-19 pandemic acted as a significant catalyst in this regard. With lockdowns, travel restrictions, and an overarching emphasis on physical distancing, the need for digital solutions, including in mental health and therapeutic services, surged dramatically. Within this backdrop, it becomes imperative to explore how therapists and clients in Pakistan navigated this sudden pivot to screen-to-screen interactions (11, 13). The rationale for focusing on Pakistan lies in its unique intersection of rapidly growing digital infrastructure and cultural nuances that shape therapeutic interactions.

Thus, the essence of this study is to delve deep into the multifaceted dimensions of digital therapeutic interventions within Pakistan, especially during and post the pandemic era. The objective is twofold: firstly, to illuminate the experiences, benefits, challenges, and adaptations of therapists as they transitioned to digital platforms; and secondly, to understand the varied therapist and their clients’ perspectives, feedback, and expectations from this mode of therapy. By doing so, this research aims to provide a comprehensive understanding, grounded in real experiences, that could guide future practices, policies, and digital strategies in the realm of therapy and counselling, not just in Pakistan but in similar contexts globally.

## 2 Literature review

### 2.1 Historical context of therapeutic interventions

Historically, therapeutic interventions have emphasized face-to-face interaction, as this direct form of communication has been deemed invaluable for fostering a genuine human connection (1, 14). Such interactions have been pivotal in creating a bond of trust between therapists and patients, grounded in their mutual presence. Being in the same space allowed therapists to not only hear the concerns of their patients but also to observe non-verbal cues such as facial expressions, posture, and gestures, which often provide deeper insights into a patient’s state of mind (15).

In earlier eras, the framework for therapy was built around the premise that healing occurred in dedicated therapeutic spaces. The therapist’s office became a sanctuary, a safe space where patients could freely express their vulnerabilities, fears, and hopes (16, 17). This physical space’s ambiance, often designed to be calm and serene, played a role in the therapeutic process (16). Over time, various therapeutic methods and approaches evolved, but the central tenet remained consistent: the physical presence of both therapist and patient was indispensable.

### 2.2 Rise of digital interventions globally and in Pakistan

With the advent of the 21st century, the world witnessed a high concern for mental health issues (18) along with a surge in digital revolution that permeated almost every facet of human life. Healthcare, not wanting to be left behind, began leveraging technological advancements to provide services more efficiently and broadly (19, 20). This shift was particularly pronounced in the field of therapeutic interventions (21, 22). What started as telehealth initiatives, primarily in the West, slowly transformed into full-fledged digital therapeutic platforms. These platforms provided therapy via video conferencing, text messaging, and even augmented reality (22).

Pakistan, a country with an increasing youth population and rapid technological adoption, saw a parallel rise in mental health awareness (10, 23). While traditional therapeutic practices remained dominant, there was an evident interest in and acceptance of digital therapeutic platforms, especially among the younger demographics (13). However, challenges unique to Pakistan—like limited internet penetration in rural areas, linguistic and cultural diversity, and socioeconomic disparities—affected the uniform adoption of these digital interventions.

### 2.3 Previous studies on digital counseling: benefits, challenges, and future implications

In recent times, the digital psychotherapy approach has gained prominence, especially in light of global events like the COVID-19 pandemic. This transformation of psychotherapeutic interventions from manual to electronic based (web or smartphone application) has been studied extensively (24).

In one of the pioneering investigations into Computer- and Internet-Based Psychotherapy Interventions, Taylor and Luce (22) highlighted the promising potential of computer and internet modalities in optimizing the cost-effectiveness of psychological interventions. Their study underscored the comparable efficacy of computer-assisted therapy to traditional face-to-face consultations, particularly for managing anxiety disorders and depression. Despite the promising outcomes, they noted the scarcity of research in this sphere at the time (22).

Andrews et al. (24) highlighted the efficacy of digital therapeutic interventions, noting that both face-to-face Cognitive Behavioral Therapy (CBT) and Internet-based CBT (iCBT) show comparable outcomes. Remarkably, iCBT required significantly less therapist time and displayed good acceptability and adherence. Such findings indicate that iCBT can serve as a potent tool in addressing anxiety and depressive disorders (24).

Bisen and Deshpande assessed the effectiveness of Internet-based psychotherapeutic interventions (IBTIs). They found comparable results to face-to-face methods, emphasizing their cost-efficiency and accessibility. However, they also highlighted challenges, including symptom exacerbation and significant dropout rates. But the authors also found some issues and negative effects in IBTIs related to deterioration of symptoms as well as high rate of abandonment and the frustration caused by technical problems (14).

Weightman (25) illuminated the challenges faced by patients in remote areas, emphasizing the potential of digital psychotherapy in addressing these issues. Through several randomized controlled trials, he concluded that digital interventions, particularly cognitive-behavioral therapy, were as effective as traditional face-to-face methods.

Studies by Harrer et al. (26) and Brog et al. (6) underscored the significance of digital interventions in alleviating depressive symptoms among university students and adults, respectively. Harrer’s research also pointed to the non-stigmatizing nature of digital psychotherapy, enhancing treatment utilization among the target audience.

Sriati et al. (27) further explored the effectiveness of digital psychotherapy in addressing college students’ psychological problems during the pandemic. Their comprehensive review advocated the combined use of digital media and video conferencing to optimize therapeutic outcomes.

Moghimi et al. (28) took a slightly different angle, examining the benefits of digital mental health interventions for Public Safety Personnel and Correctional Workers. Their systematic review categorized the advantages into accessibility, structure, therapist engagement, proactive interventions, and engagement via interpersonal relationships.

Costescu et al. (29) provided an encompassing perspective on Digital Mental Health (DMH) interventions. While acknowledging their efficacy, they emphasized some issues like co-designing with end-users, ensuring user engagement, and being mindful of costs and usability. They also advocated for more inclusive and diverse future research (29).

Prescott et al. (30) analyzed a cohort of adults (N=1,852) using digital mental health platforms during the COVID-19 period. Their findings suggest digital care, notably with therapist or coaching input, enhances well-being and alleviates depressive symptoms. Importantly, telecoaching emerged as the most cost-effective approach (30).

Thanetnit (8) explored the implications of digital technologies for mental healthcare in Thailand amidst the pandemic. The author recognized their potential augmentation to traditional methods but also underscored concerns about privacy and security (8).

Lastly, Bond et al. (31) presented both the merits and challenges of digital transformation in mental health services. While they emphasized the 24/7 availability and anonymity digital platforms offer, they also raised concerns about app quality, ethical challenges, and the pitfalls of over-relying on technology.

As digital counseling and psychotherapy have gained significant traction, particularly amidst the challenges posed by the COVID-19 pandemic. Several studies have elucidated the various benefits, challenges, and implications of this evolving therapeutic approach. Andrews et al. (24) highlighted that internet-based Cognitive Behavioral Therapy (iCBT) is not only as effective as its traditional counterpart but also requires substantially less therapist time. Weightman (25) emphasized the accessibility of digital psychotherapy, especially in remote areas, stressing its potential in bridging the healthcare gap caused by a dearth of specialized face-to-face services. Such interventions, as Harrer et al. (26) indicated, are notably effective for university students, demonstrating their potential in addressing the high prevalence of depression in this demographic. This was supported further by Sriati et al. (27), who found a range of digital interventions, including apps and video conferencing, to be effective in managing student mental health during the pandemic. Additionally, the non-stigmatizing nature of digital platforms enhances its appeal, making it a more accessible mode of therapy for many (6, 26). However, the terrain isn’t without its challenges. While digital interventions offer an array of benefits, such as 24/7 accessibility (31), they also face issues of user retention, quality assurance, and potential over-reliance on technology as well as privacy and security concerns as highlighted by (8). Ethical concerns, such as the perceived reduction in care quality when shifting from human to digital interaction, also loom large. The synthesis of this literature underscores the vast potential of digital therapeutic interventions but also signals the need for addressing the inherent challenges to maximize their efficacy and reach.

### 2.4 Gap in the literature: what this study aims to contribute

While there is a substantial body of research exploring digital therapeutic interventions, literature has often taken a more global or Western-centric approach (32, 33). This perspective, while valuable, often fails to encapsulate the unique challenges and nuances of countries like Pakistan, with its distinct cultural, linguistic, and socioeconomic dynamics.

Moreover, while many studies detail the theoretical and practical applications of digital therapies, there’s a dearth of research delving into patients’ perceptions, experiences, and satisfaction levels, particularly in non-Western contexts. The potential impact of societal norms, stigma surrounding mental health, and technological accessibility on the adoption and effectiveness of these interventions in Pakistan remains largely uncharted territory.

This research aims to bridge this gap. By focusing on Pakistan, it intends to shed light on the benefits, challenges, and future implications of digital therapeutic interventions in a context that has yet to be thoroughly explored. Through this lens, it hopes to provide valuable insights that can influence both local and global strategies, policies, and recommendations in the ever-evolving landscape of digital mental health care.

## 3 Methodology

In this section, the methods employed to gather, examine, and interpret data for this study are elucidated. The research leans into a qualitative framework, chosen for its adeptness in extracting the intricate layers of individual experiences, especially in the realm of digital therapeutic interventions. This methodological approach is detailed in the subsequent sections, highlighting the rationale for participant selection, the specifics of data collection via in-depth interviews, and the systematic process of data analysis employing Braun and Clark’s thematic analysis (34).

### 3.1 Overview of the qualitative approach

Qualitative research, at its core, seeks to interpret phenomena by delving into the intricacies of human experiences, beliefs, and emotions. Unlike quantitative research that quantifies phenomena into numerical data, the qualitative paradigm embraces the subjectivity and richness of individual narratives (35). In the context of this study on digital therapeutic interventions, a qualitative approach was deemed suitable for several reasons. Firstly, it enables the uncovering of nuanced personal experiences and challenges that users face with digital therapy. Secondly, it allows for the exploration of underlying motivations, apprehensions, and preferences that might influence the adoption and effectiveness of such interventions.

### 3.2 Selection of participants: rationale and process

Selecting participants who can offer valuable insights is critical to the success of qualitative research. For this study, the selection process was both deliberate and systematic through purposive sampling. The participants were chosen based on their experience or association with digital therapeutic interventions.

For this study, participants were sought primarily from mental health institutions based in Rawalpindi and Islamabad. Inclusion criteria for the study was those counselors and therapists who have active involvement in providing digital counseling and therapeutic interventions; having experience of offering digital therapeutic services for more than six months; a clinical work background of over one year; falling within the age bracket of 25 to 60 years and having affiliation with a mental health organization.

The study spanned a duration of three months, from February 2022 to April 2022, during which time in-depth interviews were conducted with a total of 12 counselors and therapists. Of these professionals, 60% (n=7) were men, while 40% (n=5) were women. They hailed from mental health organizations within Rawalpindi and Islamabad, with an average age of 35 years (range: 25–60). Their educational backgrounds varied from Master’s degrees to PhDs. Only those who met the specified inclusion and exclusion criteria were considered eligible participants.

The rationale for this criterion-centric selection was rooted in the study’s objective to understand the real-world intricacies of using digital therapeutic platforms. By targeting individuals who have undergone or are familiar with these interventions, the research aimed to gather firsthand accounts and experiences.

### 3.3 Development of interview guideline

The interview method was chosen based on literature supporting its use for investigating such research questions (36). The interview guide was developed following the evidence that suggests the guidelines should include questions to address the study’s basic objectives and focus, as the primary criteria (37). Questions were developed to assess participants’ perspectives and preferences for digital therapeutic tools and explore their perceptions of the challenges and problems, benefits and future outlook, and additional insights.

A panel of experts, including the primary authors, developed the interview guide. A committee of three practicing clinical psychologists was invited to assess the face validity of the questions. The interview guide was pre-tested on a pilot sample (n = 5) to evaluate its feasibility, questioning sequence, and the dynamics involved in using prompts and extending the conversation.

In accordance with the criteria for evaluating the qualitative research (38), it was ensured that the interviewer thoroughly understood the subject matter and the specific context of the study. The researcher (author 3), who conducted the interviews possesses a background in psychological assessment, psychometry, clinical psychology, and counseling. Additionally, this author received training from the primary authors regarding the conduction of the interview in context of the present study.

### 3.4 Data collection: in-depth interviews

Those shortlisted were approached with an introductory email, and upon expression of interest, they were provided with detailed information about the research’s purpose, scope, and their rights as participants. This was followed to the stage, where they were requested to sign the consent form.

In-depth, semi-structured telephonic interviews were the primary mode of data collection. These interviews were audio-recorded with participants’ consent for later transcription and analysis ensuring the maintenance of ethical standards, particularly in preserving anonymity and ensuring data security. No personally identifiable information was gathered. Data collection was continued until saturation was obtained.

### 3.5 Data Analysis

Post data collection, the transcribed interviews underwent a detailed analysis using the acclaimed Braun and Clark’s 6-step thematic analysis method. This approach ensures the extraction of coherent, relevant themes from qualitative data.

This methodical approach to analysis ensures the findings are both grounded in participants’ experiences and framed within the broader context of digital therapeutic interventions.

## 4 Findings

The core of this research hinges on the insights unearthed through our rigorous thematic analysis. Using thematic analysis, the vast information gleaned from the in-depth interviews was distilled into a set of discerning themes. These themes, both intricate and overarching, represent the multifaceted experiences, perceptions, and opinions of the participating therapists and counselors. Each theme, in its essence, captures a unique facet of digital counseling, encompassing both the challenges and benefits experienced by mental health professionals in the realm of online therapeutic services. Through this section, readers will be introduced to each of these themes in detail, unraveled and elucidated to shed light on the broader landscape of digital counseling in the context of our study area. The meticulous breakdown and interpretation of these themes serve not only as a window into the lived experiences of our participants but also as a beacon for future research and practice in the ever-evolving domain of digital therapy.

**Table 1** presents the themes extracted from the interviews. The themes are categorized based on benefits it provides, issues it has and challenges it presents, for the purpose of clarity.

**Table 1:** Extracted themes related to broader categories of benefits, issues, and challenges of online therapeutic care model.

Sr #	Themes
Benefits	
**1**	Expanding Reach and Accessibility
**2**	Client Experience: Comfort and Accessibility
**3**	Future of Digital Interventions
Issues	
**4**	Digital Security and Confidentiality
**5**	Diverse Feedback, Emotions, and Expectations
**6**	Adjustment to Digital Platforms
Challenges	
**7**	Interpersonal Challenges
**8**	Adapting Therapeutic Techniques

The categorization of themes into benefits, issues, and challenges provides a structured way to understand and evaluate the multifaceted impacts of digital counseling. Each theme serves to shed light on a particular facet of the digital therapy experience.

Among the benefits, “Expanding Reach and Accessibility” underscores the eradication of geographical barriers, democratizing access to therapy. The theme of “Client Experience: Comfort and Accessibility” spotlights the flexibility digital platforms offer, allowing therapy in personal comfort zones. As we look ahead, “Future of Digital Interventions” suggests the evolving role of digital interventions in therapeutic practices, emphasizing that their relevance isn’t fleeting but foundational.

However, every innovation brings its own set of concerns. “Digital Security and Confidentiality” accentuates the data security concerns intrinsic to online platforms. Differing experiences give rise to “Diverse Feedback, Emotions, and Expectations,” illuminating the varied reactions and the potential misalignment of expectations between therapists and clients. Furthermore, the transition to online hasn’t been smooth for everyone, as highlighted by “Adjustment to Digital Platforms “, showcasing the initial challenges in assimilating digital counseling.

Lastly, challenges in the digital therapeutic environment surface through “Interpersonal Challenges” — an exploration of the complexities in fostering genuine connections online. The theme “Adapting Therapeutic Techniques” addresses the need for therapists to recalibrate their traditional techniques for the digital environment, underscoring the call for continuous training in this evolving domain. Each of these themes together provides a holistic understanding of the landscape of digital counseling in the present context.

### 4.1 Defining and naming themes

#### 4.1.1 Benefits

##### 4.1.1.1 Expanding reach and accessibility — geographical liberation in therapy

This theme extracted from how digital counseling during the pandemic has allowed therapists to reach clients without the limitations traditionally imposed by geographical distance. The digitalization of therapy during the pandemic served as a bridge when physical mobility was constrained, making therapy more accessible to diverse populations. Therapists could reach clients from varied geographical locations and backgrounds, sometimes expanding their client base and offering flexible hours due to the elimination of commute. That’s why this theme has been referred as “*Geographical Liberation in Therapy*”.

##### 4.1.1.2 Client experience: comfort and accessibility

This theme reflects on how clients have received digital therapy – from the comfort of personal spaces to accessibility. Digital therapy as expected is accessible from anywhere both for the therapist as well as client. The shift from the traditional therapy room to online platforms introduced a new dynamic in the therapist-client relationship. While the venue change impacted the therapeutic experience for many, responses varied widely. Some clients found comfort in familiar surroundings, while others missed the traditional setting.

##### 4.1.1.3 Future of digital interventions — futuristic

This theme ponders on therapists’ and clients’ perceptions regarding the lasting role and impact of digital interventions post-pandemic. While the COVID-19 pandemic accelerated its adoption, its future is shaped by efficacy, acceptance, and evolving societal needs. Both therapists and clients are mostly satisfied with its usage and want its adoption, but again some clients long for physical sessions when there is no hindrance. Hence, this theme has been renamed as “Futuristic”.

#### 4.1.2 Issues

##### 4.1.2.1 Digital security and confidentiality — guarding confidentiality in the digital realm

This theme addresses concerns and measures regarding the security and privacy of online therapy sessions. Questions regarding data security, potential breaches, and technological vulnerabilities were paramount. Ensuring client data’s safety was central, and therapists adopted various measures and tools to address these concerns. This leads to renaming the theme as “*Guarding Confidentiality in the Digital Realm*”.

##### 4.1.2.2 Diverse feedback, emotions and expectations

This theme explores the variety of client feedback, which ranged based on factors like age, tech-savviness, and personal preferences. With the shift to digital therapy, therapists received a spectrum of feedback from their clientele. Responses and preferences varied, underscoring the diversity in client expectations. For instance, differences were noticeable between younger and older clients in their perceived challenges and benefits of digital therapy. Some clients feel detached from the therapist when doing digital therapy sessions and happy in physical sessions.

##### 4.1.2.3 Adjustment to digital platforms — from novelty to norm

This theme highlights the initial resistance or unfamiliarity therapists as well as clients felt while adjusting to the digital mode of counseling. The pandemic-enforced rapid transition to online platforms represented a significant shift for therapists. Beyond just adopting a new tool, it was about integrating technology into a profession steeped in human connection. The initial hurdles and the learning curve were evident, but over time, strategies emerged to assimilate the digital format effectively. Therefore, this theme was renamed as “*From Novelty to Norm*”.

#### 4.1.3 Challenges

##### 4.1.3.1 Interpersonal challenges

This theme investigates the unique challenges faced in building rapport and fostering connection in an online setting. The introduction of a digital screen between the therapist and client sometimes affected the therapeutic relationship’s depth and quality. While technology bridged the physical gap, challenges arose in establishing the closeness inherent in face-to-face sessions. The nuances of reading non-verbal cues and adapting methods to maintain connection became crucial.

##### 4.1.3.2 Adapting therapeutic techniques

This theme looks into how therapists had to modify or adapt certain therapeutic techniques to suit the online mode. Not all traditional therapeutic methods translate seamlessly to the digital environment. Constraints and possibilities of the digital format prompted therapists to evolve their techniques, ensuring the therapy’s core remained intact. This is still a challenge to be dealt with.

However, some therapists suggest that training and professional development must be going on in facilitating a smooth transition to digital counseling. It will bridge the gap between traditional methods and their digital equivalents.

## 5 Discussion

### 5.1 Dynamics of digital therapeutic care

The findings from the thematic analysis reveal a nuanced understanding of the digital counselling landscape, enriched by the diverse experiences of both therapists and clients during a time of global upheaval.

A dominant theme that emerged was the “*Geographical Liberation in Therapy*.” The pandemic accelerated the digital transformation in many sectors, and therapy was no exception. As one therapist noted, “*The absence of geographical limitations is significant, and clients could reach out even from remote areas*.” This geographical democratization in the therapeutic realm resonates with previous studies. Harrer et al. emphasized the potential of Computers and Internet-based programs to provide cost-effective psychological assessment and treatment (26). The findings not only corroborate this but delve deeper into its implications. The elimination of geographical barriers means more than just cost savings (30); it signifies an expansion in the diversity of clients a therapist can cater to like a therapist stated that “*the reach is phenomenal. I’ve had clients from parts of Pakistan I hadn’t even heard of, and it’s been enlightening*”, thus potentially enriching the therapeutic process with varied cultural and regional perspectives. The existing evidence on the potential benefits of digital care highlights its affordability and accessibility, noting that it can help address mobilization challenges and potentially reduce the burden of hospitalization (39). Among potential benefits, a recent cross-sectional survey of healthcare professionals (N = 860) reported that telemedicine bridged the access gap and perceived to be potentially more advantageous, even when considering potential barriers to its impact on recovery (40).

Furthermore, the theme “*Client Experience: Comfort and Accessibility*” sheds light on the evolving dynamics of the therapist-client relationship in the digital age. A poignant snippet from a client’s transcript reads, “*the most evident benefit is accessibility. Digital platforms opened doors for individuals who were hesitant or found it logistically challenging to attend physical sessions*” and “*reaching clients who previously couldn’t access therapy due to location constraints has been the most significant advantage*”. While some clients revealed in the newfound comfort of home-based sessions, others expressed a sense of loss. One therapist shared, “*Younger clients appreciate the flexibility, while older ones sometimes find it challenging to navigate the technology and longing for the warmth of physical sessions*.” These divergent experiences are reminiscent of Bisen and Deshpande (14) findings, which highlighted the advantages of online interventions in terms of cost-effectiveness and accessibility but also acknowledged potential pitfalls such as symptom deterioration and technical issues.

As we look forward, the “*Future of Digital Interventions - Futuristic*” theme becomes paramount. A therapist mused, “*I believe the digital mode will continue in parallel with face-to-face sessions. It has shown us an alternative way, especially beneficial for reaching distant or hesitant clients*.” The sentiment is echoed by another participant who stated, “*While I value traditional methods, the potential of digital counselling is undeniable. I believe a hybrid model will emerge post-pandemic*.” Indeed, the emergent trend towards digital interventions, propelled by the pandemic, seems likely to continue, shaped by evolving societal needs, technological advancements, and therapeutic innovations. These outcomes also resonate with broader literature like Thanetnit (8) who stated digital therapies as a future trend in the area. The futuristic vision of digital healthcare demands an integrated healthcare model that aligns with technological and quality standards of healthcare services (39), seamlessly incorporated into the public health infrastructure.

However, as with all technological advancements, there come inherent challenges. The theme “*Digital Security and Confidentiality – Guarding Confidentiality in the Digital Realm*” underscores this. A therapist shared his concerns, “*Using secure platforms and educating clients on their part in ensuring privacy is essential. Yet, sometimes, clients themselves might not prioritize their own privacy, which is concerning*” and “*I’m wary of data breaches. I’ve tried to educate myself on the best practices and insist on using secure platforms. Digital security has been a prime concern for me*”. Such concerns echo Thanetnit (8) emphasis on the pressing need to address privacy and security issues in digital health interventions.

Also, the evolution in the therapeutic process was evident in themes like “*Diverse Feedback, Emotions and Expectations*” and “*Adjustment to Digital Platforms – From Novelty to Norm*.” The transition wasn’t seamless for all. As one therapist revealed, “*For over a decade, I had worked closely with my clients, feeling their emotions and resonating with their sentiments in a physical space. The digital world felt detached initially. But as days turned into weeks, I began to appreciate the nuances*” and “….*one fundamental challenge was deciphering non-verbal cues, the unsaid words that often reveal more than spoken ones sometimes we capture more from the body language and face readings. The screen sometimes made it challenging to capture those subtle indications*”. Similarly, clients’ experiences varied based on age, technological adeptness, and personal preferences. Feedback from a therapist about a younger client’s elucidated this: “*Younger clients appreciate the flexibility, while older ones sometimes find it challenging to navigate the technology*.” Contrastingly, older clients are more tilted towards physical sessions like a therapist stated about his experience that, “*many from the older generation yearned for the warmth of face-to-face interactions*.” This echoes the sentiments captured by Bisen and Deshpande (14), who noted the emergence of new psychological symptoms and challenges inherent in Internet interventions.

Therapies are more than just therapeutic sessions. Therapists study the emotions and feeling so their clients and attach them emotionally. A therapist detailed her feelings about the digital sessions that “*The challenge is ensuring that the emotional connect remains intact. Sometimes, it’s harder to read emotions or comfort a distressed client through a screen. Also, some clients express a feeling of detachment in the virtual setting*”. Although different clients of different ages have different opinions and feedback, digital therapy is a new normal same as other digital innovations and will be here. The resilience and adaptability displayed by therapists and clients alike align with findings from Prescott et al. (30). They demonstrated the efficacy of digital therapy during the unprecedented challenges of the pandemic, emphasizing the comparative benefits and cost-effectiveness of such platforms.

The research also dived deep into “*Interpersonal Challenges*.” The digital realm, with all its advantages, sometimes posed obstacles in establishing the warmth and rapport traditionally fostered in face-to-face sessions. One therapist poignantly remarked, “*Building trust through a screen is a different challenge. Building rapport is sometimes more challenging. The nuances of body language and energy in a room, which often guide therapy, are harder to interpret*.” Another therapist stated the same challenge that “*Building rapport, especially with new clients, takes longer and demands more effort. Sometimes, interpreting non-verbal cues becomes difficult.*” This resonates with Costescu et al. (29) insights, which urged the need for co-designing digital interventions with end users, emphasizing the importance of maintaining the essence of human connection.

Lastly, the theme “*Adapting Therapeutic Techniques*” brought forth an intriguing dimension of the shift to digital counseling. Therapists were challenged to modify traditional techniques to suit the constraints and opportunities of the digital medium. “*I think many therapeutic techniques I used in-person had to be modified for the digital space*,” shared one therapist. Another statement by a therapist profoundly highlighted that “*Digital interventions are here to stay. We, as practitioners, need to refine our approach and harness its potential fully*”. This sentiment aligns with studies on the implications of digital technologies in mental health care, underscoring the need for ongoing training and professional development to bridge the gap between traditional and digital methods (8, 40).

In synthesizing this discussion, the interplay between the benefits, issues, and challenges of digital therapeutic interventions becomes palpably clear. The themes, such as “Geographical Liberation in Therapy” and “Client Experience,” highlight the transformative potential of digital interventions in democratizing mental health access. At the same time, themes like “Guarding Confidentiality in the Digital Realm” and “Diverse Feedback, Emotions, and Expectations” foreground the nuanced complexities that come with this digital shift. Moreover, “Adjustment to Digital Platforms” and “Interpersonal Challenges” spotlight the resilience and adaptability required in navigating this new therapeutic terrain, and the critical necessity of “Adapting Therapeutic Techniques” underscores the ongoing evolution in the field. As we compare the findings against the broader literature, it’s evident that the digital therapy landscape, while filled with promise, necessitates thoughtful integration of technology with the profound human connection inherent to therapy. As the domain of digital mental health continues to evolve, it becomes imperative to continuously reflect, adapt, and innovate, ensuring that therapeutic interventions remain effective, accessible, and deeply resonant with those they seek to aid.

### 5.2 Sociocultural considerations within the Pakistani context

Within the Pakistani context, the findings of this study reveal both the transformative potential and the nuanced challenges of digital therapeutic interventions. Understanding these findings requires an appreciation of Pakistan’s unique sociocultural landscape. Pakistan, with its vast rural areas and rapidly growing urban centers, has long struggled with unevenly distributed healthcare resources. The theme of “Geographical Liberation in Therapy” is particularly salient in this regard, suggesting that digital therapy offers a means to bridge the urban-rural divide in mental health access.

The “Client Experience” theme resonates deeply with Pakistan’s youth, a majority of whom are tech-savvy and constantly connected online. Digital therapy, in this sense, might cater better to their needs, considering their increasing comfort with virtual interactions. Yet, it’s essential to recognize the generational differences in tech adoption. Themes like “Diverse Feedback, Emotions, and Expectations” emphasize the variability in digital therapy’s reception, reflecting broader societal divides between younger generations and their elders, who might be less familiar or comfortable with online platforms.

Privacy concerns, as brought up in “Guarding Confidentiality in the Digital Realm,” carry significant weight in a society where the stigma surrounding mental health remains potent. The fear of personal information leakage can be more pronounced given the close-knit nature of many Pakistani communities, where individuals’ reputation and family honor are highly valued. The theme of “Adjustment to Digital Platforms,” too, is underscored by the limited technological infrastructure in parts of the country and the unfamiliarity of some therapists and clients with digital tools.

“Interpersonal Challenges” in the online therapy context become all the more pronounced considering the deep-rooted cultural importance of face-to-face interactions in Pakistani society. Often, non-verbal cues, gestures, and physical presence play a crucial role in communication, and their absence or dilution in a digital format can pose challenges. Furthermore, adapting therapeutic techniques, as echoed in the theme “Adapting Therapeutic Techniques,” takes on an added dimension in Pakistan. It’s not just about translating face-to-face therapeutic methods to an online environment but ensuring they remain culturally appropriate and sensitive.

When these findings are compared to broader global literature, many similarities and differences emerge. While the world over, the pandemic has accelerated the adoption of digital therapeutic practices, the particularities of the Pakistani context — its cultural nuances, technological disparities, and societal values — shape its own unique narrative. Like many other countries, Pakistan grapples with issues of digital security, varied feedback based on demographics, and the challenges of building rapport online. Yet, the emphasis on family honor, societal reputation, and the profound importance of face-to-face interactions gives Pakistan’s experience its distinct flavor. As digital therapeutic interventions continue to evolve globally, it’s imperative to ensure they’re tailored to fit the sociocultural fabric of the communities they serve as in the words of a therapist “*Even as we inch towards normalcy, I believe digital counseling has carved its niche. It won’t replace traditional methods but will exist alongside, offering clients a choice. The long-term implication is a more flexible, adaptable, and expansive therapeutic landscape, bridging urban-rural divides and reaching those who earlier had limited access*”. In the case of Pakistan, this means a careful, thoughtful blending of technological advancements with deep-rooted cultural values.

### 5.3 Implications for therapy and counselling practices

The surge of digital therapeutic interventions and our findings from the thematic analysis have profound implications for the future of therapy and counselling practices in Pakistan.

#### 5.3.1 Inclusivity and Accessibility

One of the most significant takeaways is the potential for digital therapy to democratize access. Considering Pakistan’s vast and diverse terrain, with its mix of urban centers and remote areas, digital counselling can become a beacon of hope for those residing in regions previously underserviced by mental health professionals. Therapists based in cities like Lahore or Karachi could potentially service clients from rural Sindh or Balochistan, breaking barriers of distance and ensuring that mental health services reach those who need them most.

#### 5.3.2 Cultural Sensitivity in Digital Modalities

Digital platforms will need to be tailored not just to the technological needs but also the cultural sensitivities of the Pakistani population. This means considering language (providing services in Urdu, Punjabi, Sindhi, Pashto, and others), understanding societal norms and taboos, and being aware of the particular challenges faced by individuals in different regions or from different communities within the country.

#### 5.3.3 Training and Infrastructure

As underscored by our theme “Adjustment to Digital Platforms”, there is a pressing need for extensive training. Therapists must be equipped not just with the skills to use digital platforms, but also with the knowledge to adapt their therapeutic techniques to this new modality. Moreover, considering the technological disparities in various parts of Pakistan, there’s a need to bolster the digital infrastructure, ensuring stable and secure internet connections, and perhaps introducing low-bandwidth solutions for regions with limited connectivity.

#### 5.3.4 Addressing Stigma and Privacy Concerns

In the Pakistani context, the concerns regarding privacy and confidentiality are amplified by cultural norms and societal expectations. Digital platforms must ensure top-notch security measures to protect client data. Additionally, widespread campaigns might be necessary to educate the populace about the safety and efficacy of digital therapy, thereby reducing associated stigma.

#### 5.3.5 Tailored Therapeutic Approaches

The themes “Interpersonal Challenges” and “Adapting Therapeutic Techniques” emphasize the need to revisit and revise therapeutic techniques for the digital age. Therapists might need to develop new strategies to establish rapport, read non-verbal cues in an online setting, and ensure that the therapeutic connection remains robust even across a digital medium.

As digital therapy continues to embed itself in Pakistan’s mental health landscape, the onus is on therapists, policymakers, and technology providers to ensure that this transition is both seamless and sensitive to the country’s unique socio-cultural nuances. Embracing digital therapy doesn’t just mean adopting a new tool; it means reshaping the therapeutic experience to align with Pakistan’s evolving needs and challenges.

## 6 Limitations of the study

While this study has provided valuable insights into the realm of digital health care interventions in the Pakistani context, there are certain limitations that warrant acknowledgment. Firstly, the sample size, while diverse, may not entirely capture the vast heterogeneity of the Pakistani population in terms of ethnicities, socio-economic statuses, and varying levels of digital literacy. This implies that some perspectives, especially those from the most marginalized groups, might be underrepresented.

Secondly, the reliance on self-reported data can introduce potential biases. Participants might have been influenced by social desirability, presenting their experiences in a manner they deem more socially acceptable rather than their genuine feelings or experiences. This could especially be the case in a culture where discussing mental health might still be considered taboo in certain circles. The study did not include clients as a separate sample, but the data obtained from therapists indirectly reflects the clients’ perspectives, particularly concerning comfort, accessibility, and issues related to the therapist-client relationship.

In light of these limitations, while the findings are significant and contribute to the burgeoning field of digital therapy in Pakistan, they should be interpreted with a degree of caution. Future research endeavors could aim to address these limitations, offering an even more comprehensive understanding of the landscape.

## 7 Conclusion and future research

The exploration of digital therapeutic interventions, particularly in the unique sociocultural context of Pakistan, has unveiled a breadth of insights that underscore the dynamic interplay between technology, therapy, and cultural norms. This study illuminated how digitalization, while acting as a bridge during times of restricted mobility such as the COVID-19 pandemic, also introduced novel benefits, challenges, and issues. For instance, the “Geographical Liberation in Therapy” theme emphasized the transformative power of digital platforms in transcending traditional geographical barriers, making therapy accessible to a wider demographic. Yet, themes like “Interpersonal Challenges” and “Guarding Confidentiality in the Digital Realm” highlighted areas of potential concern that need addressing for the broader acceptance and efficacy of digital therapy.

Looking forward, as Pakistan and other similar regions become more digitally integrated, the trajectory of digital therapeutic interventions seems promising. However, for this trajectory to be optimally beneficial, there’s a need to delve deeper into certain areas. Future studies could focus on understanding the long-term impacts of digital therapy, comparing its efficacy with traditional face-to-face interventions in various therapeutic contexts. Investigating the role of digital therapeutic tools tailored specifically for Pakistani or similar populations, bearing linguistic, cultural, and sociological nuances in mind, can also be a significant area of exploration.

From a practical standpoint, recommendations emerging from this study emphasize the need for robust digital infrastructure, training therapists to adapt traditional therapeutic techniques to online formats, and devising strategies to foster deep interpersonal connections in a digital realm. Policymakers should prioritize the integration of digital literacy within therapeutic training modules, ensuring that both therapists and clients are equipped to navigate the digital therapeutic landscape. Stakeholders, especially those in the tech industry, might consider collaborating with therapists and mental health professionals to develop platforms and tools that cater specifically to the unique challenges and opportunities that digital therapy presents in Pakistan.

While the landscape of digital therapeutic interventions in Pakistan is still evolving, it holds immense potential. As technology advances, it’s crucial to ensure that its integration into therapy is done thoughtfully, keeping the cultural, social, and individual needs of the clients at the forefront. As digital healthcare becomes more integrated into Pakistan’s mental healthcare system, therapists, policymakers, and technology providers must address the country’s specific needs and challenges including mental health awareness, technological access, technological advancements, training of professionals, and the cultural sensitivity while dealing clients with diverse sociocultural backgrounds.

## Data Availability

The original contributions presented in the study are included in the article/supplementary material, further inquiries can be directed to the corresponding author/s.
